# An unusual case of foreign body aspiration mimicking cavitary tuberculosis in adolescent patient: Thread aspiration

**DOI:** 10.1186/1824-7288-38-17

**Published:** 2012-05-11

**Authors:** Erkan Cakir, Emel Torun, Zeynep Seda Uyan, Ozge Akca, Omer Soysal

**Affiliations:** 1 Bezmialem Vakif University, Medical Faculty, Department of Pediatric Pulmonology, Istanbul, TURKEY; 2 Bezmialem Vakif University, Medical Faculty, Department of Pediatrics, Istanbul, TURKEY; 3 Kocaeli University, Medical Faculty, Department of Pediatric Pulmonology, Kocaeli, TURKEY; 4 Bezmialem Vakif University, Medical Faculty, Department of Thoracic Surgery, Istanbul, TURKEY; 5 Bezmialem Vakif University, Medical Faculty, Department of Pediatric Pulmonology, Adnan Menderes Bulvarı, 34093, Fatih/ISTANBUL, TURKEY

**Keywords:** Foreign body aspiration, Thread, Flexible bronchoscopy, Child, Pediatric

## Abstract

Foreign body aspiration continues to be a serious problem in childhood and adolescent period with significant rate of morbidity and rarely mortality. Half of the foreign body aspiration cases have no history of aspiration. The main foreign bodies inhaled are food fragments and different kinds of metallic objects. A 12-year-old girl was referred to the pediatric pulmonology department for chronic cough and hemoptysis. She had persistent infiltration and cavitary lesion mimicking cavitary tuberculosis. There was no contact history with tuberculosis in her family and acid resistant bacillus was not found in the sputum examination. Flexible bronchoscopy was performed for persistent infiltration and hemoptysis and inflamed thread was found in right lower lobe bronchus. This is the first case of thread inhalation mimicking cavitary tuberculosis in an adolescent patient.

## Background

Foreign body aspiration (FBA) is an important cause of persistent respiratory problems in childhood [1,2]. The main foreign bodies inhaled are nuts, seeds, fragments of toys in infant age; sharp, metallic objects such as needles, toothpicks, safety pins in toddlers’ age and early childhood period; and blow dart and darting pin in school-aged children [3-6]. FBA is diagnosed easily with presentation of typical clinical history of aspiration and atelectasis or hyperlucency on chest radiography. Unusual and misleading cases especially without aspiration history present with asthma like symptoms such as chronic cough and wheezing, recurrent or persistent pulmonary infiltrations, bronchiectasis and atelectasis [7-10]. This is an example of misleading case, without aspiration history, presented with chronic cough, hemoptysis, persistent pulmonary infiltrations, and cavitary lesion mimicking tuberculosis.

### Case presentation

**Figure 1:**
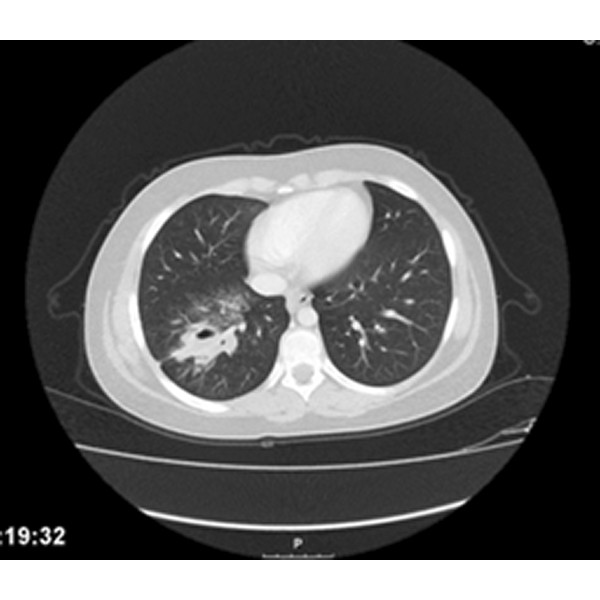
Cavitary lesion mimicking tuberculosis on chest computed tomography.

**Figure 2:**
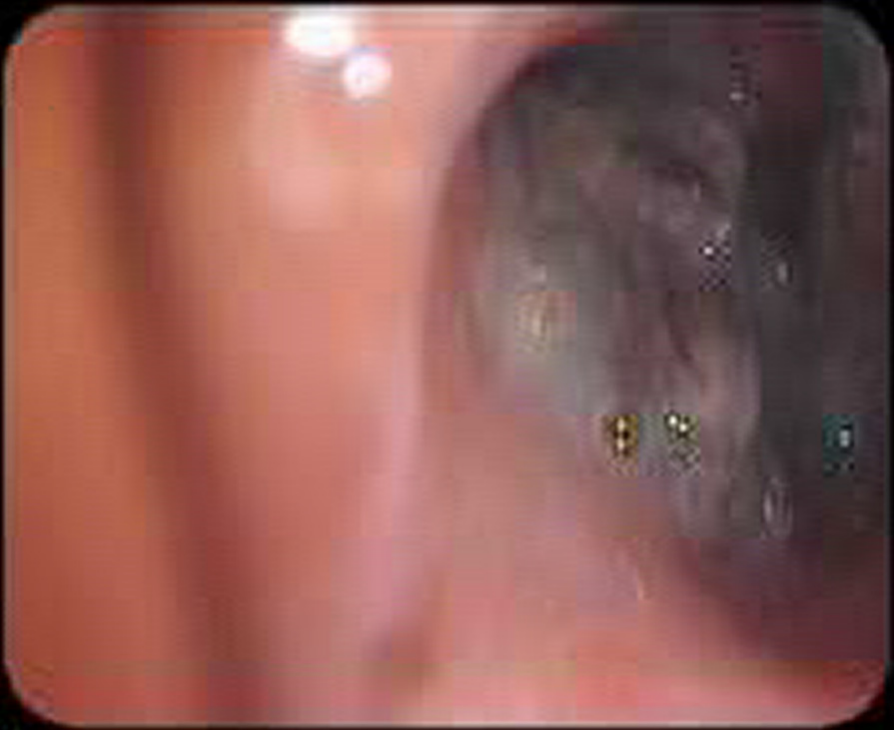
Bronchoscopic appearance of thread aspiration.

**Figure 3:**
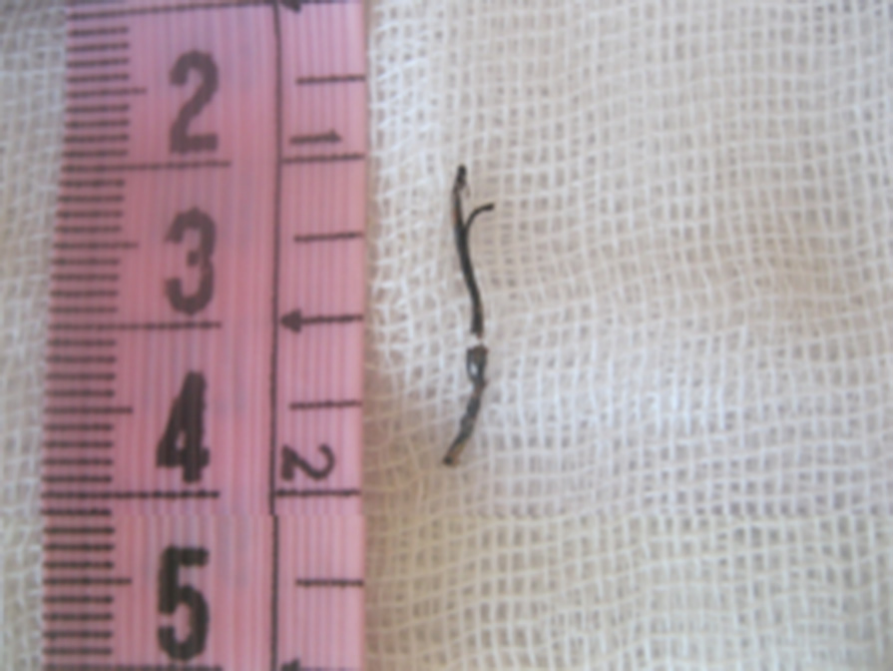
Aspirated thread.

A 12-year-old girl was referred to the pediatric pulmonology department for chronic cough, recurrent pulmonary infections for one year and episodic hemoptysis for the period of last nine months. She had no weight loss and night sweats but the productive cough became persistent and she had multiple bouts of antibiotic therapy with the diagnosis of nasopharyngitis or bacterial pneumonia. The symptoms had become much worse over the period of the last one month before she attended to our hospital with bloody sputum and purulent cough. There was no history of foreign body aspiration and contact with tuberculosis. On examination, there was no respiratory distress but had diminished breath sounds in the right thorax and crackles on the same side. The other systemic examination was normal. Chest X-ray revealed pneumonia on the right side, and cavitary lesion with infiltration was detected on the computerized tomography (CT) mimicking cavitary tuberculosis (Figure 1). Acid resistant bacillus was not found in the sputum examination. Flexible bronchoscopy was performed for persistent infiltration and hemoptysis. There was significant mucus accumulated and inflamed foreign body was found in right lower lobe bronchus with granulation tissue around the foreign body (Figure 2). Inflamed thread was removed successfully with rigid bronchoscopy (Figure 3). After removal of the foreign body, the symptoms resolved rapidly and the non-specific antibiotherapy was stopped after 2 weeks.

## Discussion

It is an unusual FBA presentation mimicking cavitary tuberculosis in an adolescent patient with long duration of respiratory symptoms and recurrent pulmonary infections on the same side of the lung even with the absence of a history of choking. Foreign body aspiration is a common medical emergency with serious airway obstruction and sudden respiratory distress occurs immediately in complete obstruction. If the obstruction is incomplete foreign body becomes lodged, progressive respiratory symptoms such as chronic cough, wheezing, hemoptysis, pneumonia and atelectasis can develop and the diagnosis is usually delayed. Chest radiographs are commonly performed in children with suspected FBA, and may show unilateral or lobar hyperlucency, localized atelectasis, and localized pulmonary infiltrate. However, a normal radiograph could not exclude FBA [2,9,10]. Our patient is an example of delayed diagnosis and misinterpreted as pneumonia and cavitary tuberculosis.

The type of the foreign body depends on cultural, social and economic factors and eating habits. Children younger than 3 years old have considerable risk of foreign body aspiration because of the tendency of using their mouths to explore their surroundings. At this stage of development, the main objects aspirated are vegetable originated seeds, peanuts and toy parts [3,7,8]. Also in our country safety pins ingestion among 4 months -2 years old children were reported [4]. Aspiration of sharp, metallic objects such as needles, toothpicks, safety pins in toddlers” age and early childhood period were also reported [4]. In school aged or adolescent period, the most common material aspirated are blow dart, thumbtack, darting pin, and headscarf needles [3,5,6]. Our patient had aspirated thread, an unusual material that was not reported in adolescent period before.

As a result, FBA is one of the life threatening emergency that may happen at any age. The misleading cases without aspiration history present with recurrent or persistent pulmonary symptoms. Bronchiectasis and atelectasis can be seen in diagnostic delayed cases as usual but cavitary lesion mimicking tuberculosis is an atypical presentation [2,9]. If the diagnosis is delayed and symptoms and signs are not specific for any disease, flexible bronchoscopy is needed to evaluate directly in such cases.

### Consent

Written informed consent was obtained from the parents of the patient for publication of this case report and any accompanying images. A copy of the written consent is available for review by the Editor-in-Chief of this journal.

## Abbreviations

(CT), Computerized tomography; (FBA);, Foreign body aspiration.

## Competing interests

All authors declare that they have no conflict of interest.

## Authors’ contributions

EC evaluated the patient, performed flexible bronchoscopy, and prepared the manuscript. ET and ÖA participated in the sequence alignment and drafted the manuscript. ZSU evaluated the patient and helped to draft the manuscript. OS performed rigid bronchoscopy and removed the foreign body. All authors read and approved the final manuscript.
